# HIV-1 Tat-induced diarrhea evokes an enteric glia-dependent neuroinflammatory response in the central nervous system

**DOI:** 10.1038/s41598-017-05245-9

**Published:** 2017-08-10

**Authors:** Giuseppe Esposito, Elena Capoccia, Stefano Gigli, Marcella Pesce, Eugenia Bruzzese, Alessandra D’Alessandro, Carla Cirillo, Alessandro di Cerbo, Rosario Cuomo, Luisa Seguella, Luca Steardo, Giovanni Sarnelli

**Affiliations:** 1 Department of Physiology and Pharmacology, “La Sapienza” University of Rome, Rome, Italy; 2 Department of Clinical Medicine and Surgery, Section of Gastroenterology, University of Naples “Federico II”, Naples, Italy; 3 Department of Translational Medical Science, Section of Pediatrics, University of Naples “Federico II”, Naples, Italy; 4 Laboratory for Enteric Neuroscience (LENS), TARGID, University of Leuven, Leuven, Belgium; 5 Department of Biomedical Science, “G. D’Annunzio” University, Chieti, Italy

## Abstract

Despite the effectiveness of combined anti-retroviral therapy, human immunodeficiency virus (HIV) infected-patients frequently report diarrhea and neuropsychological deficits. It is claimed that the viral HIV-1 Trans activating factor (HIV-1 Tat) protein is responsible for both diarrhea and neurotoxic effects, but the underlying mechanisms are not known. We hypothesize that colonic application of HIV-1 Tat activates glial cells of the enteric nervous system (EGCs), leading to a neuroinflammatory response able to propagate to the central nervous system. We demonstrated that HIV-1 Tat-induced diarrhea was associated with a significant activation of glial cells within the colonic wall, the spinal cord and the frontal cortex, and caused a consistent impairment of the cognitive performances. The inhibition of glial cells activity by lidocaine, completely abolished the above-described effects. These observations point out the role of glial cells as putative effectors in HIV-1 Tat-associated gastrointestinal and neurological manifestations and key regulators of gut-brain signaling.

## Introduction

The involvement of the gastrointestinal tract is a major clinical feature in patients with acquired immunodeficiency syndrome (AIDS) and represents one of the main causes of morbidity and mortality related to the disease^1^. Despite the effectiveness of the combined anti-retroviral therapy, diarrhea is frequently reported by human immunodeficiency virus (HIV) infected-patients, with the viral HIV-1 Trans activating factor protein (Tat) being identified as one of the main pathogenic mechanism^2^. In over fifty percent of HIV-infected adults, cognitive problems and significant neuropsychological deficits have been demonstrated as well, even in the absence of HIV-replication^3^. These observations suggest that most of the HIV-related pathological conditions may be related to HIV-1 Tat toxicity, rather than to the virus *per se* ^4^.

In the gut, HIV-1 Tat induces diarrhea by altering enterocytes intracellular calcium concentration, and this results in the secretory diarrhea and the cellular damage^5^. However, HIV-1 Tat also affects the function of the enteric nervous system (ENS) and this further amplifies the intestinal dysfunction^6^. *In vitro* experiments indicate that HIV-1 Tat is able to increase the excitability of cultured enteric neurons and to stimulate the release of proinflammatory cytokines, probably through the activation of enteric glia cells^7, 8^. Conversely, in the CNS, a more direct involvement of glial cells in mediating HIV-1 Tat-induced effects has been observed^9^; indeed, HIV-1 Tat-overexpression in astrocytes results in a significant upregulation of the glial fibrillary acidic protein (GFAP), with astrocytosis and an increased release of proinflammatory cytokines^9^.

These observations point out the role of glial cells as pivotal targets and potential effectors in HIV-1 Tat-associated gastrointestinal and neurological manifestations^4, 6, 8^.

In particular, enteric glial cells (EGCs) are involved in the maintenance of gut homeostasis by their ability to reinforce the epithelial barrier function^10–13^, and, more recently, they have been identified to regulate intestinal inflammatory responses^14–16^ and to mediate host-pathogen interactions^17^. The involvement of EGCs in mediating the effects of HIV-1 Tat in the colon has never been investigated so far.

Collectively, these results provide the rationale for our hypothesis that is to demonstrate, in a rat model of intracolonic administration, the involvement of EGCs in HIV-1 Tat-induced diarrhea and to verify: i) if and how the activation of the enteric glia cells modulates the diarrhea, ii) if EGC-activation is localized at the intestinal level, or it is associated with a signaling to the CNS iii) to characterize the pathway by which HIV-1 Tat signaling propagates from the periphery to the brain, and iv) to correlate these events with cognitive impairment.

## Results

### Intracolonic HIV-1 Tat administration induces diarrhea and activates an enteric glia-mediated neuroinflammatory response

**Figure 1:**
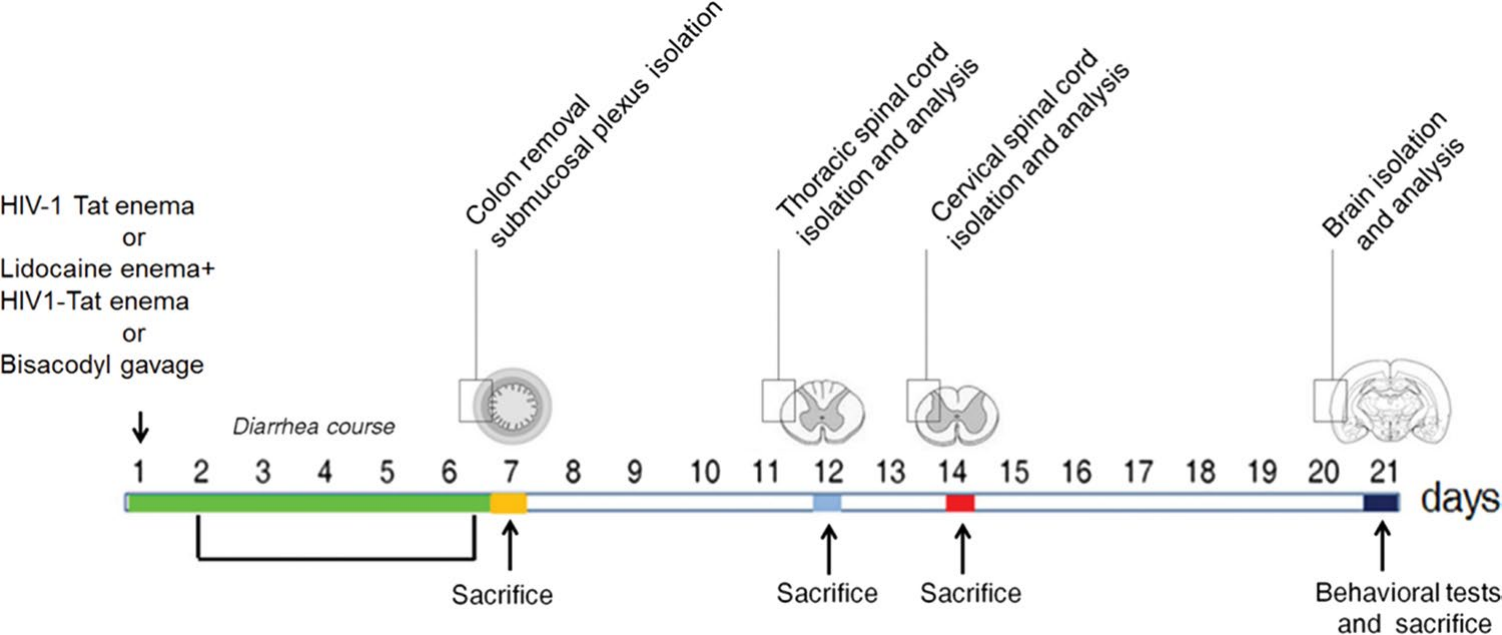
Schematic representation of rats-induced diarrhea. Diagram showing the induction of diarrhea by a single intracolonic administration of HIV-1 Tat, alone or in the presence of lidocaine, or by a single dose of bisacodyl, and the time schedule for measurements on enteric, or central nervous system glia cells.

**Figure 2:**
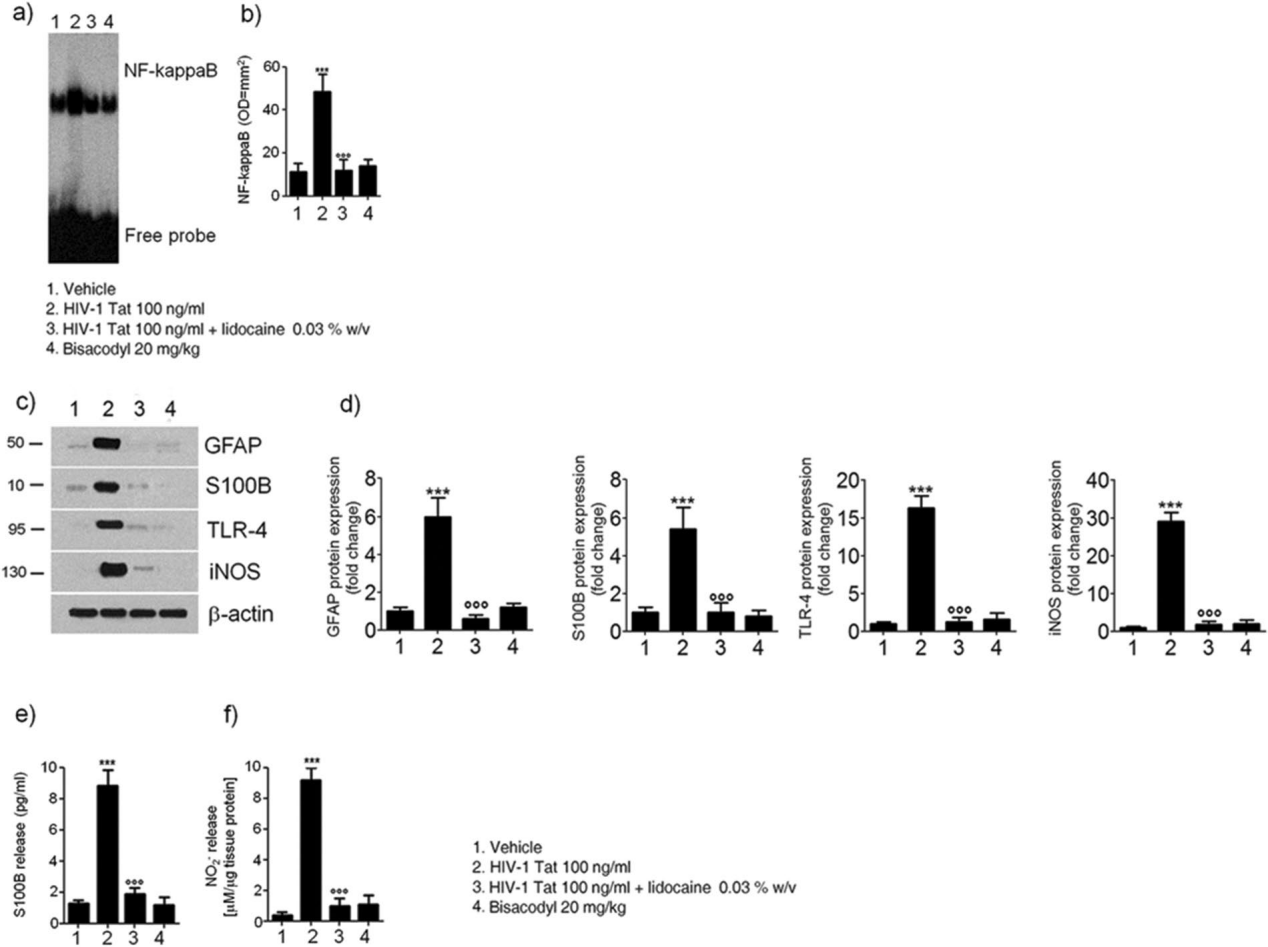
(**a**,**b**) EMSA analysis showing that intracolonic administration of HIV-1 Tat (100 ng/ml) induced a marked increase of NF-kappaB expression in EGCs nuclear extracts versus vehicle group. The administration of lidocaine significantly reduced HIV-1 Tat-induced NF-kappaB activation, whereas bisacodyl failed to induce any significant effect on NF-kappaB activation. (**a**) The panel shows representative NF-kappaB activation complex bands and (**b**) their densitometric quantification (OD = optical density in mm^2^). (**c**,**d**) HIV-1 Tat treatment caused a marked increase of GFAP, S100B, TLR-4 and iNOS protein expression in submucosal plexus lysates, as compared to vehicle group and this effect was significantly inhibited by lidocaine; to note that bisacodyl also failed to induce any significant effect. (**c**) The panel shows representative immunoreactive bands of analyzed proteins and (**d**) their respective levels expressed as fold change. (**e**,**f**) In the medium of submucosal plexi lysates obtained from HIV-1 Tat treated rats a significant increase of NO_2_^−^ and S100B was observed in comparison with vehicle group and such effect was counteracted by lidocaine; again, bisacodyl had no effect on treated animals. Results are expressed as mean ± SEM; ***p < 0.001 vs all other groups; °°°p < 0.001 vs HIV-1 Tat group; n = 6 for each group.

The study protocol is summarized in the Fig. 1. Intracolonic application of HIV-1 Tat induced an acute onset diarrhea lasting for 7 ± 3 days; the severity and duration of diarrhea were significantly inhibited by the concomitant application of lidocaine (Supplementary Figure 1). EMSA analysis showed that nuclear NF-κB was significantly increased in the submucosal plexi-lysates of HIV-1 Tat treated animals (Fig. 2a and b), and this was associated with a significantly higher expression of EGCs’ markers like GFAP, S100B, TLR-4 and iNOS (Fig. 2c and d); similarly, the release of S100B and nitrite production were significantly increased (Fig. 2e and f). Lidocaine application prevented HIV-1 Tat induced NF-κB activation, down-regulated the expression of GFAP, S100B, TLR-4 and iNOS, and reduced the release of S100B and nitrite; no significant differences were instead observed between bisacodyl-treated and control animals (Fig. 2a–f).

**Figure 3:**
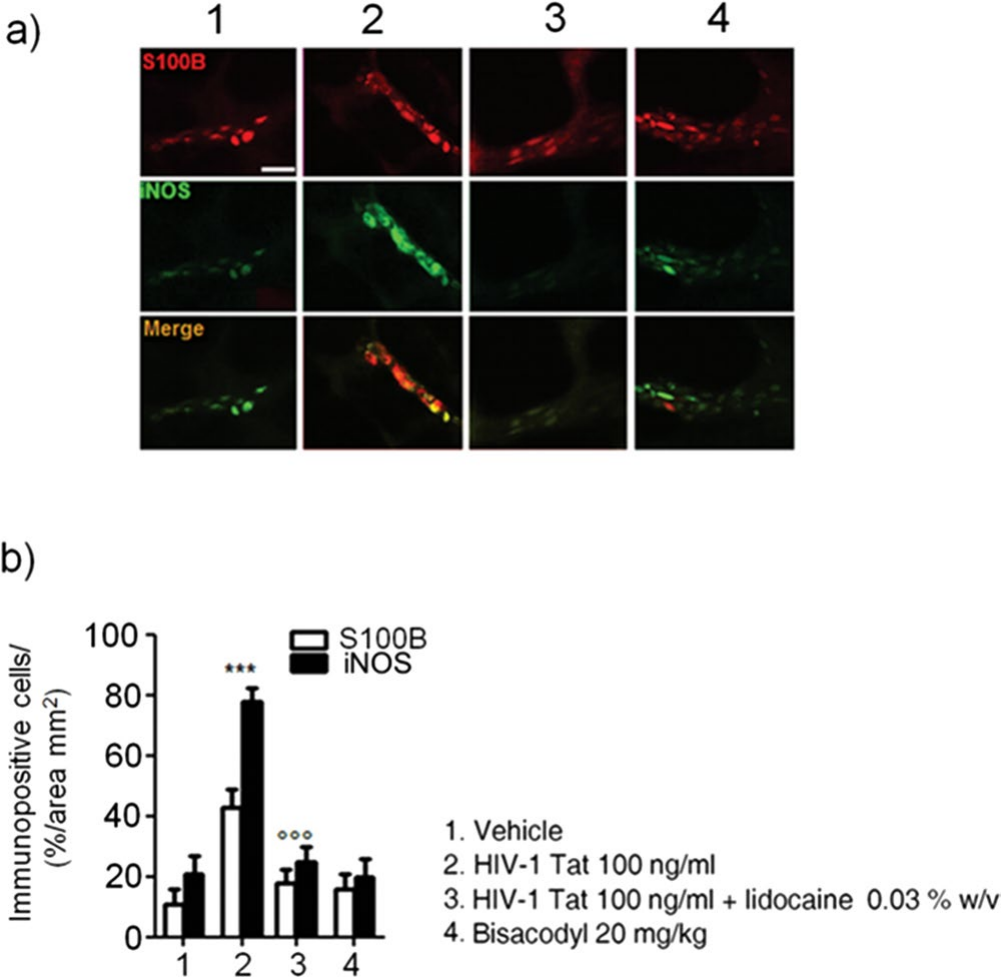
Intracolonic administration of HIV-1 Tat (100 ng/ml) yields to a marked activation of submucosal plexus-EGCs, as shown by the immunofluorescence analysis showing a significant increase of S100B and iNOS protein co-expression. (**a**) The panel shows iNOS (green) and S100B (red) immunoreactivity with (**b**) the respective quantification of iNOS (filled bars) and S100B (open bars) expression in the EGCs; both lidocaine pretreatment and bisacodyl administration failed to significantly affect S100B/iNOS expression. Results are expressed as mean ± SEM; ***p < 0.001 vs all other groups; °°°p < 0.001 vs HIV-1 Tat group. Scale bar: 20 μm.

To further demonstrate that the effects induced by HIV-1 Tat were specific and evoked the activation of EGCs, immunofluorescence analysis carried on isolated submucosal plexi revealed an up-regulation of S100B and iNOS, and both were significantly inhibited by lidocaine treatment (Fig. 3); no significant changes in S100B and iNOS protein expression were instead observed in rats with bisacodyl-induced diarrhea. These results suggest that HIV-1 Tat-induced diarrhea was at least partially mediated by the activation of submucosal EGCs that, once activated, mediate a local neuroinflammatory response.

### HIV-1 Tat-induced EGCs activation triggers the upregulation of GFAP and S100B in spinal cord and frontal cortex glial cells through the expression of Connexin43

**Figure 4:**
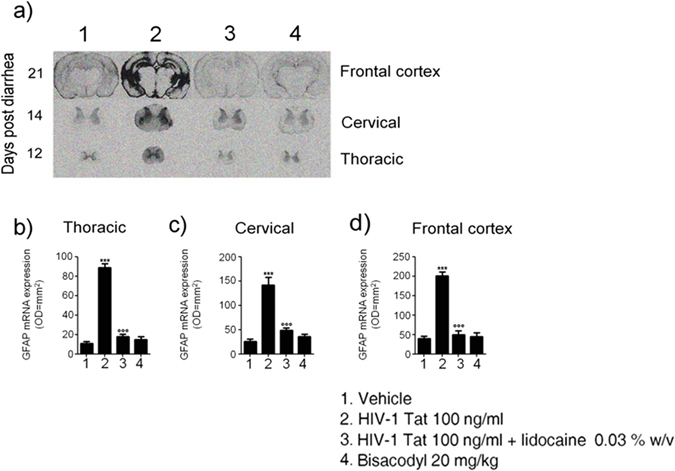
(**a**) *In situ* hybridization analysis of thoracic and cercival spinal cord, and frontal cortex showing GFAP mRNA expression at day 12, 14 and 21 after diarrhea induction, respectively. (**b**–**d**) Quantitative analysis revealed that administration of HIV-1 Tat protein caused a significant increase of GFAP mRNA expression in all the analyzed areas compared, as compared to both vehicle or lidocaine group, while bisacodyl yields to no significant change. (Results are expressed as mean ± SEM; ***p < 0.001 vs all other groups; °°°p < 0.001 vs HIV-1 Tat group; OD = optical density in mm^2^; n = 5 for each group).

In order to verify whether HIV-1 Tat-induced glia activation was localized at the intestinal level or associated with a signaling to the CNS, we evaluated the late onset activation of glia cells at different levels of the spinal cord and the frontal cortex. After 12, 14 and 21 days from HIV-1 Tat administration, the expression of GFAP mRNA was significantly increased in the thoracic, cervical spinal cord and brain frontal cortex, respectively (Fig. 4a and b).

**Figure 5:**
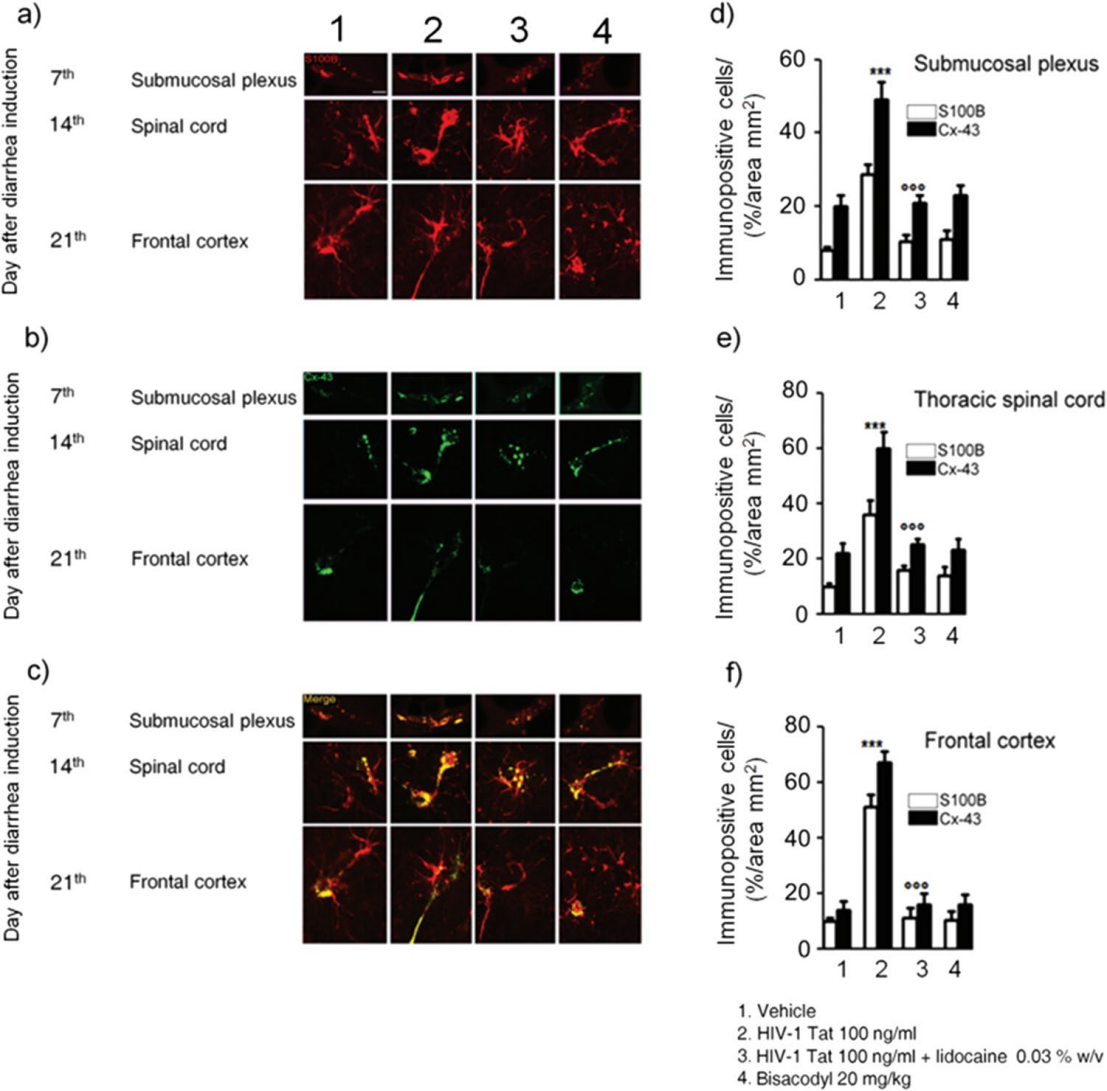
(**a**–**c**) Intracolonic administration of HIV-1 Tat caused a marked increase of Cx-43 (green) protein expression in S100B-positive cells (red) in the submucosal plexus, spinal cord and frontal cortex, respectively. (**d**–**f**) Quantitative analysis revealed that administration of lidocaine significantly reduced Cx-43 expression on S100B-positive cells of HIV-1 Tat group, whereas bisacodyl did not produce any change in Cx-43 expression compared to vehicle group (S100B (open bars) and Cx-43 (filled bars) expression in the analyzed areas. ***p < 0.001 vs all other groups; °°°p < 0.001 vs HIV-1 Tat group. Scale bars: 20 μm; n = 6 for each group.

Such activation was characterized by a time-dependent significant overexpression of Connexin43 (Cx43), likely mediating cell-to-cell connection. Indeed, immunofluorescence analysis showed that in HIV-1 Tat-treated animals a higher percentage of Cx43 and S100B expressing cells was observed in the submucosal plexus, the spinal cord and the frontal cortex as compared to control rats, respectively (Fig. 5). Interestingly, when HIV-1 Tat was administered in the presence of lidocaine, both the upregulation of GFAP mRNA and the expression of Cx43/S100B in all the analyzed areas were significantly reduced (Figs 4 and 5); similar findings were observed in bisacodyl-treated rats (Figs 4 and 5). These results suggest that modulation of HIV-1 Tat-induced diarrhea by lidocaine inhibits enteric glia activation and prevents cell-to cell signaling from the gastrointestinal tract to the CNS.

### HIV-1 Tat-induced gliosis is associated with a neuroinflammatory response in the spinal cord and brain cortex

**Figure 6:**
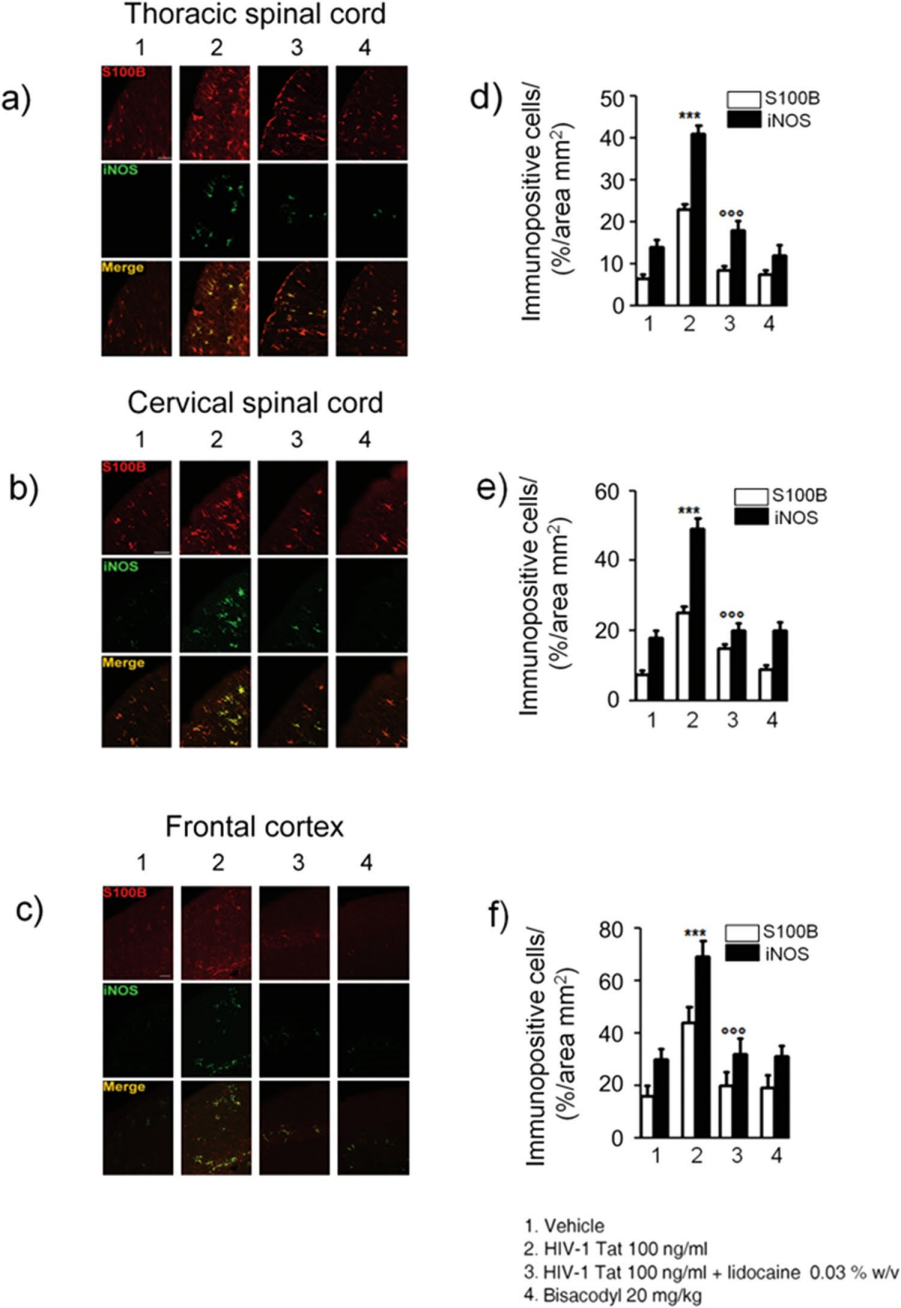
Intracolonic administration of HIV-1 Tat induced glial activation in the (**a**) thoracic and (**b**) cervical spinal cord and (**c**) frontal cortex at day 12, 14 and 21 after diarrhea induction, respectively. (**a**–**c**) Immunofluorescence analysis showed that iNOS (green) and S100B (red) co-expression was increased in the spinal cord and frontal of HIV-1 Tat treated rats. (**d**–**f**) Quantitative analysis showed that HIV-1 Tat-induced upregulation of iNOS (filled bars) and S100B (open bars) was significantly inhibited by lidocaine treatment. Results are expressed as mean ± SEM; ***p < 0.001 vs all other groups; °°°p < 0.001 vs HIV-1 Tat group. Scale bars: 100 μm; n = 6 for each group.

**Figure 7:**
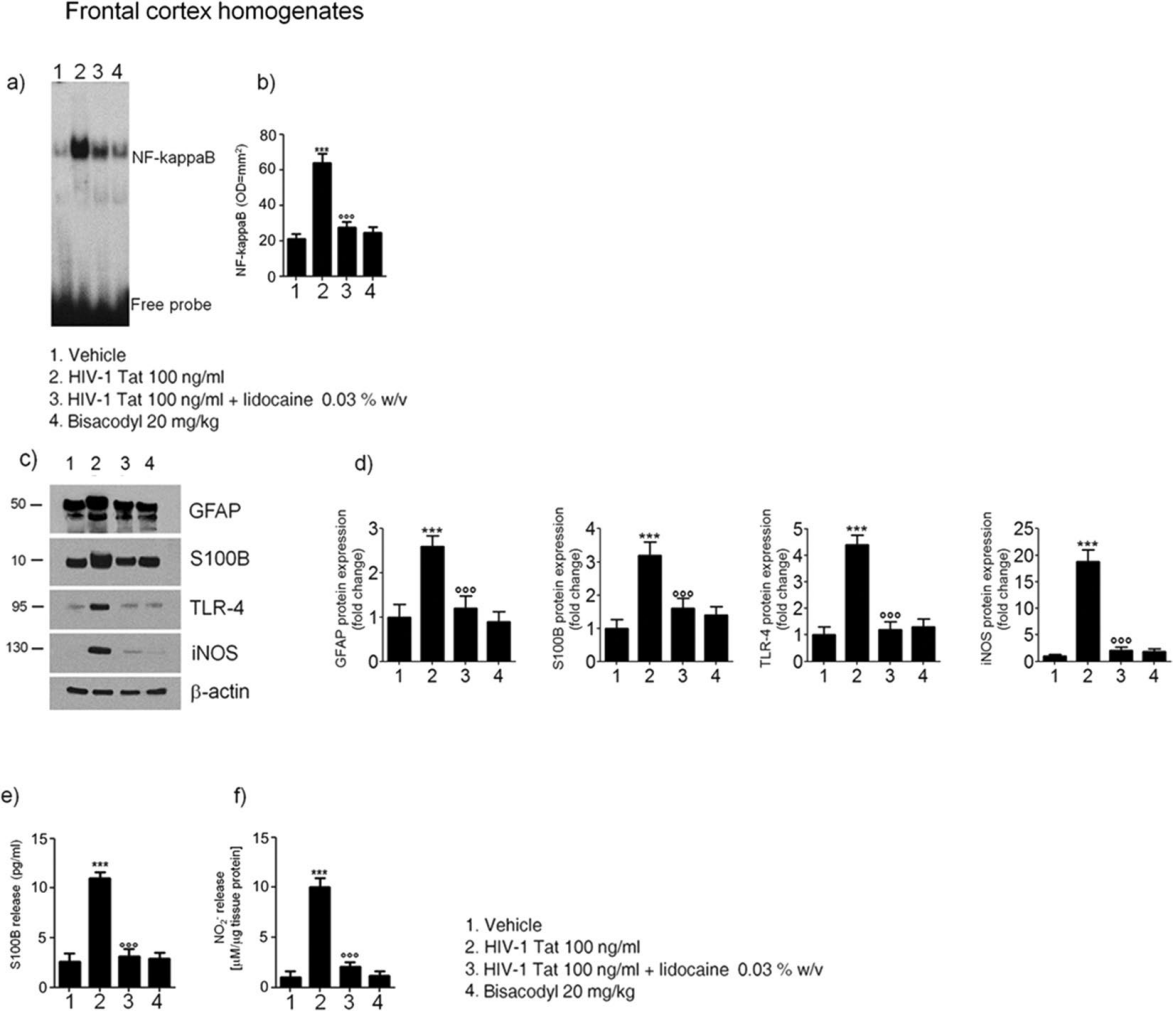
Effect of HIV-1 Tat treatment on NF-kappaB activation in the nuclear extracts of frontal cortex and astrocytes activation. (**a**) The panel shows representative NF-kappaB activation complex bands in the different groups of rats. (**b**) The quantitative analysis revealed that intracolonic HIV-1 Tat administration yields to a significant increase of NF-kappaB, as compared to vehicle, lidocaine, or bisacodyl groups (OD = optical density in mm^2^). (**c**) HIV-1 Tat caused a marked increase of GFAP, S100B, TLR-4 and iNOS protein expression in the frontal cortex homogenates of treated rats. (**d**) Quantitative analysis reveled that HIV-1 Tat induced a significantly higher expression of GFAP, S100B, TLR-4 and iNOS, than lidocaine, or bisacodyl groups. (**e**,**f**) In the medium of frontal cortex homogenates deriving from HIV-1 Tat group a significant increase of NO_2_^−^ and S100B was also observed as compared to the other groups. (Results are expressed as mean ± SEM; ***p < 0.001 vs all other groups; °°°p < 0.001 vs HIV-1 Tat group; n = 6 for each group).

Similarly to what observed in the submucosal plexus, immunofluorescence analysis revealed that, 12, 14 and 21 days after HIV-1 Tat administration, the expression of S100B and iNOS was significantly increased in the thoracic and cervical spinal cord, and in the frontal cortex, respectively (Fig. 6). Once again, application of lidocaine was able to significantly inhibit this response, while in the bisacodyl-treated animals S100B and iNOS expression was similar to control rats (Fig. 6). As late consequence of submucosal enteric and spinal cord glia activation a significant increase of NF-kappaB expression was observed in the nuclear extracts obtained from brain frontal cortex homogenates of HIV-1 Tat-treated animals (Fig. 7a and b); this increase was associated with a parallel raise of GFAP, S100B, TLR4 and iNOS expression and of S100B release and nitrite production (Fig. 7c–f).

In rats receiving lidocaine all the above-described findings were completely abolished, while bisacodyl treatment did not yield to any significant variation compared to vehicle group (Fig. 7); these results suggest that the inhibition of EGCs –HIV-1 Tat induced signaling was able to prevent the late onset activation of glial cells in the frontal cortex and to inhibit the related neuroinflammatory response.

### HIV-1 Tat-induced neuroinflammatory brain responses led to a cognitive dysfunction

In HIV-patients minimally impaired neuropsychological and behavior functions have been described even in the absence of viral replication^3^. In order to get new insights into the pathophysiological role of the intestinal-HIV-1 Tat in the decline of cognitive functions we investigated the memory skills of treated rats by the object recognition test. We found that, 21 days after HIV-1 Tat administration, there were no significant differences in the time spent exploring the novel object versus the known one, likely indicating a mild cognitive dysfunction (Supplementary Figure 2). Conversely, in rats treated by lidocaine a significant increase of the recognition index was observed, while the lack of any significant effect on memory tasks observed in the bisacodyl group indicates that the cognitive/memory dysfunction was dependent by HIV-1 Tat exposure, rather than being related to diarrhea *per se*.

## Discussion

Diarrhea is present in nearly 60–80% of HIV-infected patients, and although it is more common in third-world countries, but it is also a frequent clinical feature despite the effectiveness of combined anti-retroviral therapy^18^.

So far, the HIV-1 Tat protein has been identified as the main responsible for the mucosal damage in the gut. This viral protein induces the pro-oxidative- and pro-apoptotic-mediated disruption of epithelial cells in the colon, thus disrupting the intestinal barrier^19^. More recently, it has been described that HIV-1 Tat protein has an additional effect on the nerve part of the gut, the ENS. This direct action on the nerve system, which regulates many intestinal functions, causes abnormalities in neuronal excitability, that together with the release of proinflammatory cytokines in the intestinal milieu, contributes to gut dysfunction described in patients with HIV^6–8^.

Here we demonstrate that beside its effect on enteric neurons, HIV-1 Tat protein targets also EGCs^20^. Specifically, when applied into the colon, HIV-1 Tat triggers the activation of submucosal EGCs with the overexpression of glial proteins, namely S100B and GFAP. Activation of EGC was accompanied by the switch-on of the molecular pathway leading to the induction and release of pro-inflammatory factors, like iNOS protein and NO. These final events occur via the activation of the NF-kappaB-mediated cascade and TLR4 activation, two pathways that are linked each other during inflammation-related EGC activation^16^.

As shown in a pioneering study by Lundgren *et al*.^21^, the selective pharmacological modulation of the ENS, by using local anesthetics such as lidocaine, is able to inhibit the of rotavirus-induced diarrhea in mice. Accordingly, here we confirmed the role of the ENS in HIV-1 Tat-induced diarrhea too, since the co-administration of lidocaine reduced all the symptoms and biochemical markers indicative for secretory diarrhea.

Although we cannot definitely rule out the role of enteric neurons dysfunction in mediating this effect, the observation that, lidocaine suppresses EGC activation, is strongly supported by the inhibition of S100B and iNOS overexpression together with the suppression of TLR-4/NF-kappaB axis. Further in support of this evidence, there is the observation that when secretory diarrhea was induced by a non-immunological stimulus like bisacodyl, no significant changes in glial network and markers were observed, confirming that gliosis does not represent an unspecific feature of diarrhea, but that this is specifically involved in HIV-1 Tat-induced secretory diarrhea.

A gut-brain connection has been identified over the last years and increasing data support the hypothesis that in certain circumstances the gut may be the “entrance door” by which bacteria, prion proteins, viruses or their neurotoxic proteins may migrate to the brain to finally cause damage in the CNS^22, 23^. For example, the injection of formalin in the rat colonic wall induced c-Fos expression in the myenteric plexus, the spinal cord and the brainstem, in a retrograde way^24^, similarly, in a model of intestinal inflammation associated with the post-operative ileus, the increased expression of cycloxigenase-2 leads to cFos activation in the spinal cord via the ascending nerve pathways^25^.

In our setting we demonstrated that the local application of HIV-1 Tat by determining the activation of EGCs in the colonic submucosal plexi is able to trigger a neuroinfammatory response that propagates to the CNS. Indeed, after the induction of the diarrhea, a time dependent propagation and a significant up-regulation of GFAP mRNA and protein were observed in the thoracic and cervical spinal cord, and in the brain cortex, respectively. Further confirming the spreading of glial activation from the ENS to the CNS glia, a significant overexpression of the TLR-4/NF-kappaB pathway and an increased expression of GFAP, S100B and iNOS protein were also measured up to two weeks after the induction of the diarrhea. Interestingly, EGCs HIV-1 Tat-induced response and the related propagation from the gut to the brain were significantly blocked by lidocaine. Although we did not provide a detailed analysis of the mechanism beyond the effect of lidocaine, previous reports suggest that this may be dependent by its ability to inhibit voltage-gated Na^+^ channels on glia cells, thus preventing cells’ activation, or, alternatively, by its ability to reduce the induced glia-mediated inflammatory signaling pathways^26, 27^.

Further supporting the concept that the colonic HIV-1 Tat application represents a specific stimuli for EGCs triggering and the spread of activation of glia cells outside the gut there is also the observation that the protein was virtually absent in the CNS (see Supplementary Figure 3). Furthermore, the absence of any signs of glial activation in the brain of bisacodyl-treated rats reinforces the hypothesis that pathologic signals lifting from the enteric to the CNS glia are due to the selective priming of EGC by HIV-1 Tat protein, rather than to the diarrhea *per se*.

In the attempt to explain how EGCs and CNS astrocytes communicate during HIV-1 Tat-induced diarrhea, we tested the involvement of the gap junction protein Cx43, that is involved in cell-to cell communication, and whose expression is profoundly regulated by inflammation and dependent by intracellular Ca^2+^ ^28–30^. The expression of Cx43 in S100B-positive cells was significantly up-regulated in the submucosal plexi of HIV-1 Tat treated rats, and, more interestingly, an increased expression of Cx43 was also observed in S100B expressing glia cells of the spinal cord and frontal cortex, respectively. Again, the pretreatment with lidocaine yields to an overall and significant inhibition of HIV-1 Tat-induced Cx-43/S100B overexpression, suggesting that the inhibition of the priming stimuli in the colon is able to block the glia-mediated signaling from the gut to the central nervous system.

We finally evaluated whether the late onset of neuropathological features in the brain due to HIV-1 Tat treatment could affect the cognitive performances in our experimental models. Behavior tests showed that rats with HIV-1 Tat-induced diarrhea presented a significant worsening of mnemonic/cognitive performances, as assessed by the object recognition test. Although our study is limited because we did not evaluate the putative neurodegeneration associated with HIV-1 Tat induced glia-mediated neuroinflammation, our findings are coherent with both clinical and *in vitro* data showing a significant cognitive decline in HIV-infected even in the absence of viral replication, or an astrocytes-mediated neuronal degeneration, respectively^3^.

In conclusion, our study demonstrates that a single colonic application of HIV-1 Tat induces an acute diarrhea that is at least partially modulated by the activation of glia cells in the submucosal plexus. This local response is able to trigger and activate glia cells in the spinal cord and brain cortex through the expression of Cx43, that results in an inflammatory reaction in the brain and that is associated with a significant cognitive decline in treated rats.

## Methods

### Animals and experimental design

Eight-weeks-old Wistar male rats (Harlan Laboratories, Udine, Italy) were used for experiments. All procedures were approved by La Sapienza University’s Ethics Committee. Animal care was in compliance with the IASP and European Community (EC L358/1 18/12/86) guidelines on the use and protection of animals in experimental research. Rats were randomly divided into the following groups: vehicle, HIV-1 Tat, lidocaine plus HIV-1 Tat, and bisacodyl.

HIV-1 Tat (1–86) was chemically synthesized by a step-by-step solid phase peptide synthesis according to our previously described protocol^31^. In brief, the protein was dissolved in pyrogen-free distillated water and a volume of 400 μl of a 100 ng/ml solution of HIV-1 Tat was injected into the lumen of the rat colon, 3–4 cm proximal to anus by using a 24 gauge catheter; an equivalent volume of apyrogenic distilled water was administered into the colon of the vehicle group. In a subset of animals HIV 1-Tat was administered immediately after a 400 μl of a, 0.03% w/v solution of lidocaine hydrochloride monohydrate (Sigma-Aldrich, Milan, Italy; catalog number L5647) dissolved in sterile and pyrogen-free distilled water. Immediately after the enema, rats were kept in a vertical position for 5–10 min to avoid leakage of the instilled intracolonic solutions and animals were all held in a gentle manner to minimize any stress. In another group of animals a single dose of bisacodyl (20 mg/Kg) (Sigma-Aldrich, Milan, Italy; catalog number B1390) was administrated orally by gavage. The study protocol is summarized in the Fig. 1 and depending upon the scheduled experimental plan, animals were euthanatized and colon, thoracic and cervical spinal cord and brain were isolated to perform immunofluorescence, *in situ* hybridization and biochemical/molecular analyses as described below.

### Diarrhea evaluation

Depending on the experimental protocol, animals were separated in subgroups and placed separately in cages lined with filter paper that was changed every 2 h. The severity of the diarrhea was assessed every 2 h for 16 hours from day 1 to day 7. Frequency of defecation and number of the wet spot were recorded and compared with the score of the vehicle group. Evaluation of accumulation of intracolonic fluid was performed using the enteropooling technique according to Ateufack *et al*.^32^. Briefly, enteropooling is defined as the intraluminal accumulation of fluid into the small intestine and corresponds with the fluid already located in the lumen and excreted from the blood. Depending upon the experimental plan, the last portion of colon rats was isolated, taking care to avoid tissue rupture and loss of fluid, removing the mesentery and connective tissue. To normalize the data, fluid accumulation was expressed as follows:

$$({{\rm{W}}}_{1}-{{\rm{W}}}_{2})/{{\rm{W}}}_{2}\times {10}^{-6}$$

where W_1_ is the weight of the colon after excision and W_2_ is the weight of the colon after expulsion of its content. Water content was measured and compared with the score from the vehicle group.

### Tissue preparations

To study the effects of HIV 1-Tat-mucosal challenge on enteric glia cells, 2 cm colonic segments were used to prepare submucosal plexus by carefully removing the mucosal and the muscle layers, according to a slightly modified previously reported method^33^. On completion of the study 12 or 14 days after HIV 1-Tat administration, the left ventricle was cannulated and perfused with saline, and, after the removal of the vertebral column and the spinal cord; coded sections from spinal cervical and thoracic segments (C2–C6 and T4 to T8, respectively) were isolated and processed for biochemical or immunofluorescence assays. In a subset of animals, brain was isolated and processed for biochemical assays; part of the brain cortex was also isolated and processed to isolate astrocytes according to our previously reported methods^34, 35^.

### Protein extraction and western blot analysis

Proteins were extracted from both submucosal plexi and brain astrocytes, obtained from rats at day 7 and 21 after diarrhea induction, respectively. Cellular extracts were homogenized in ice-cold hypotonic lysis buffer to obtain cytosolic extracts and underwent electrophoresis through a polyacrilamide minigel. Proteins were transferred into nitrocellulose membrane that was saturated with non-fat dry milk and then incubated with either mouse anti-S100B (Neo-Marker, Milan, Italy; catalog number MA1-25005), mouse anti-inducible Nitric Oxide Synthase (iNOS), rabbit anti-GFAP, mouse anti-TLR4, and mouse anti-β-actin (all from Abcam, Cambridge, UK; catalog numbers ab49999, ab7260, ab30667 and ab8226, respectively). In another set of supplementary experiments, specific mouse anti-HIV-1 Tat (Biolegend, San Diego, CA, USA; catalog number MMS-116P) was used. Membranes were then incubated with the specific secondary goat anti-mouse and goat anti-rabbit antibodies conjugated to horseradish peroxidase (HRP) (Dako, Milan, Italy; catalog number P0447 and P0448). Immune complexes were revealed by enhanced chemiluminescence detection reagents (Amersham Biosciences, Milan, Italy; catalog number RPN2108). Blots were analyzed by scanning densitometry (Versadoc, Bio-Rad Laboratories) and results expressed as relative fold change and normalized on the expression of the housekeeping protein β-actin.

### Electrophoretic mobility shift assay (EMSA)

EMSA was performed to detect NF-kappaB activation in both submucosal plexi and brain astrocytes. Double stranded oligonucleotides containing the NF-kappaB recognition sequence for rats (5–CAACGG CAGGGGAATCTCCCTCTCCTT-3) were end-labelled with ^32^Pγ-ATP (Amersham, Milan, Italy). Nuclear extracts were incubated for 15 min with radiolabeled oligonucleotides (2.5–5.0 × 10^4^ cpm) in 20 ml reaction buffer containing 2 mg poly dI-dC, 10 mM Tris–HCl (pH 7.5), 100 mM NaCl, 1 mM EDTA, 1 mM dl-dithiothreitol, 1 mg/ml bovine serum albumin, 10% (v/v) glycerol. Nuclear protein-oligonucleotide complexes were resolved by electrophoresis on a 6% non-denaturing polyacrylamide gel in 1 Tris Borate EDTA buffer at 150 V for 2 hrs at 4 °C. The gel was dried and autoradiographed with an intensifying screen at −80 °C for 20 hrs. Subsequently, the relative bands were quantified by densitometric scanning with Versadoc (Bio-Rad Laboratories) and a computer programme (Quantity One Software, Bio-Rad Laboratories)^25^. P-γ-ATP was from Amersham (Milan, Italy; catalog number PB10168). Poly dI-dC was from Boehringer-Mannheim (Milan, Italy; catalog number 1219847). Oligonucleotide synthesis was performed to our specifications by Tib Molbiol (Boehringer-Mannheim).

### Nitric oxide quantification

Nitric oxide (NO) was measured as nitrite (NO_2_^−^) accumulation in submucosal plexi and brain astrocytes homogenates, by a spectrophotometer assay based on the Griess reaction. Briefly, Griess reagent (1% sulphanilamide, 0.1% naphthylethylenediamine in H_3_PO_4_) was added to an equal volume of plasma or supernatant and the absorbance was measured at 550 nm. Nitrite concentration (nM) was thus determined using a standard curve of NaNO_2_.

### Enzyme-linked immunosorbent assay for S100B

Enzyme-linked immunosorbent assay (ELISA) for S100B (Biovendor R&D, Brno, Czech Republic; catalog number RD192090100R) was carried out on both submucosal plexi and brain astrocytes lysates, according to the manufacturer’s protocol. Absorbance was measured on a microtitre plate reader. S100B level was determined using standard curves method.

### Immunofluorescence analysis

Submucosal plexi preparations were fixed for 30 minutes in ice-cold 4% paraformaldehyde, washed with PBS 1X then blocked with bovine serum albumin, and then incubated in a mixture containing a mouse anti-S100B (1:200 dil v/v; Neo-Marker, Milan, Italy; catalog number MA1-25005) and a rabbit anti-iNOS (1:100 dil. v/v; Abcam, Cambridge, UK; catalog number ab49999). Tissues were then washed (3 × 10 min) with PBS and incubated for 2 h at room temperature, with a mixture of anti-rabbit fluorescein isothiocyanate-conjugated (Abcam, Cambridge, UK; catalog number ab6717) and anti-mouse Texas Red-conjugated (Abcam, Cambridge, UK; catalog number ab6787), respectively.

Tissue sections (15 µm) of thoracic and cervical spinal cord and frontal cortex were isolated from rats at days 12, 14 and 21, respectively. Slices were fixed for 30 minutes in ice-cold 4% paraformaldehyde, washed with PBS 1X then blocked with bovine serum albumin. Sections were then stained with mouse anti-S100B (1:200 dil v/v) and rabbit anti-Cx43 (1:300 v/v; Cell Signaling Technology, Danvers, USA; catalog number 3512) or rabbit anti-iNOS antibody (1:100 v/v;). Appropriate negative controls were carried out by omitting primary antibodies. To test any non-specific antigen-binding sites, additional experiments were performed using specific isotype antibody controls (Abcam, Cambridge, UK), at the same concentration as the primary antibodies. Slices were then incubated in the dark with the proper secondary antibody: fluorescein isothiocyanate-conjugated anti-rabbit (Abcam, Cambridge, UK; catalog number ab6717) or Texas Red-conjugated anti-mouse (Abcam, Cambridge, UK; catalog number ab6787), respectively. Slices were analysed with a microscope (Nikon Eclipse 80i), and images were captured by a high-resolution digital camera (Nikon Digital Sight DS-U1).

### *In situ* hybridization

Tissue sections of thoracic and cervical spinal cord and frontal cortex were isolated from rats at day 12, 14 and 21, respectively. Sections were fixed in ice-cold 4% paraformaldehyde for 20 min, rinsed in PBS, quenched for 15 min in 1% H_2_O_2_ methanol solution, rinsed in PBS, quenched for 8 min in 0.2 M HCl, rinsed in PBS, treated with proteinase K 20 μg/ml (Roche Molecular Diagnostics, Milan, Italy; catalog number 03 115 887 001) in 50 mM Tris-HCl, 5 mM ethylene diamine tetra acetic acid (EDTA) (pH 8.0) for 10 min, rinsed in PBS, fixed in ice-cold 4% paraformaldehyde, incubated for 10 min in 0.1 M triethanolamine (pH 8.0) to which 1.2 ml acetic anhydride was added dropwise, rinsed in PBS, washed with 0.9% NaCl for 5 min, dehydrated in graded series of ethanol and air-dried. Hybridization was carried out in 100 μl of hybridization buffer containing specific sense or antisense^28^ S-labelled riboprobe for glial fibrillary acidic protein (GFAP; 70,000–100,000 c.p.m./μl). Hybridization buffer consisted of 50% deionized formamide, 20 mM Tris-HCl (pH 8.0), 0.3 M NaCl, 5 mM EDTA (pH 8.0), 10% dextran sulphate (Sigma, Milan, Italy; catalog number 51227), 0.02% Ficoll 400 (Sigma; catalog number F2637), 0.02% polyvinylpyrrolidone (PVP 40; Sigma; catalog number PVP40), 0.02% bovine serum albumin (BSA; Sigma; catalog number A2153), 0.5 mg ml^−1^ tRNA (Roche Molecular Diagnostics; catalog number 109500), 0.2 mg/ml fragmented herring sperm DNA and 200 mM dithiothreitol. Before applying to the tissue the hybridization cocktail was denatured for 2 min at 95 °C. Slides were incubated overnight at 54 °C in a humidified chamber. Four high-stringency washes were carried out at 62 °C with 5X saline sodium citrate (SSC)/0.05% Tween-20, then with 50% formamide/2X SSC/0.05% Tween-20, with 50% formamide/1X SSC/0.05% Tween-20 and finally with 0.1X SSC/0.05% Tween-20. Slides were dehydrated in graded ethanol series, air-dried and exposed to Biomax MR film (Scientific Imaging Systems, NY, USA). GFAP mRNA expression was semi-quantified by densitometric scanning of the Biomax film with a GS 700 imaging densitometer (Bio-Rad Laboratories, CA, USA) and a computer programme (Molecular Analyst, IBM, Milan, Italy).

### Object recognition test

The object recognition (OR) test is commonly used to assess the behavioral function in rodents^36, 37^. The test is carried on in two steps: a first session (acquisition) and a second session (test). The animal is faced with two similar objects during the first session, and then one of the two objects is replaced by a new object (novel) during the second session. The amount of time taken to explore the new object provides an index of the recognition memory. In our setting, the object recognition test was performed in an open-field arena of black Plexiglas (50 cm × 40 cm × 63 cm). Depending upon the experiments, and before their sacrifice, rats underwent a 8 min acquisition trial, during which the animal was placed in the open field in presence of two identical objects (namely A, that were a cube or a ball) and located at 15 cm from the arena wall (acquisition task); at the end of the exploration time, animals were returned to their respective cages for 3 hours. After the retention interval, the rats were placed back into the box and exposed to the known object A and to a novel object B for further 8 min (test task). The objects were placed in the same locations as the previous ones. The position of the novel object was randomly chosen to avoid preferences not based on novelty. Exploratory behavior was considered with the animal directing its nose toward the object closely (<2 cm) and the amount of time spent exploring each of the two objects. To measure recognition memory, a recognition index was calculated as the amount of time exploring the familiar object (TA) or the novel object (TB) in according with the formula:

$$[{\rm{TA}}\,{\rm{or}}\,\mathrm{TB}/(\mathrm{TA}+\mathrm{TB})]\times 100$$

In the acquisition and retention trials, if the exploration time was <30 s and < 15 seconds, respectively, the rats were excluded from the trial.

### Data analysis

All values are expressed as the mean ± SEM. Statistical differences were determined to be significant at P < 0.05. The specific tests used are described in the figure legends. All analyses were performed using GraphPad Prism software (GraphPad Software, Inc., CA USA). The investigators were not blinded to allocation during experiments and outcome assessment.

## Electronic supplementary material

Supplementary Information
