# Supplementary material for: HIV-1 Tat-induced diarrhea evokes an enteric glia-dependent neuroinflammatory response in the central nervous system
Source: Sci Rep. 2017 Aug 10;7:7735. doi: 10.1038/s41598-017-05245-9 (PMC5552820; doi:10.1038/s41598-017-05245-9)
Supplement: Supplementary file 1 — Supplementary Information [file 41598_2017_5245_MOESM1_ESM.pdf]

HIV-1 Tat-induced diarrhea evokes an enteric glia-dependent neuroinflammatory response in the central nervous system.

Giuseppe Esposito[1*], Elena Capoccia[1], Stefano Gigli[1], Marcella Pesce[2], Eugenia Bruzzese[3], Alessandra D'Alessandro[2], Carla Cirillo[4], Alessandro di Cerbo[5], Rosario Cuomo[2], Luisa Seguella[1], Luca Steardo[1], Giovanni Sarnelli[2*].

Supplementary Figure 1

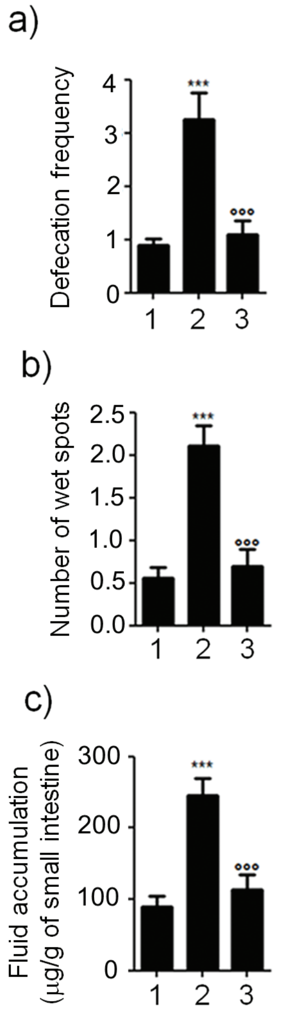

1. Vehicle
2. HIV-1 Tat 100 ng/ml
3. HIV-1 Tat 100 ng/ml + lidocaine 0.03 % w/v

Supplementary Figure 2

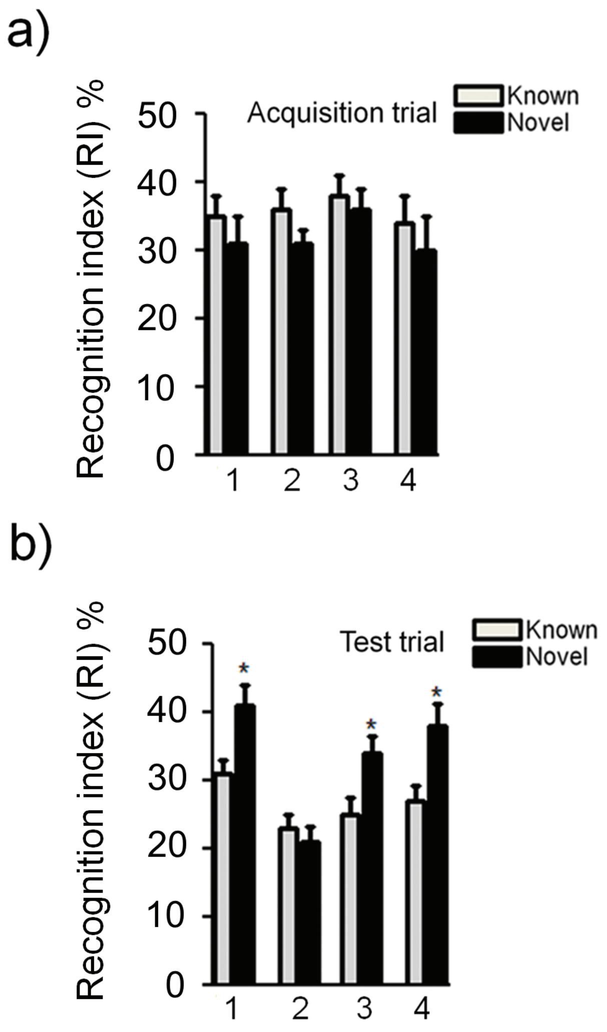

1. Vehicle
2. HIV-1 Tat 100 ng/ml
3. HIV-1 Tat 100 ng/ml + lidocaine 0.03 % w/v
4. Bisacodyl 20 mg/kg

Supplementary Figure 3

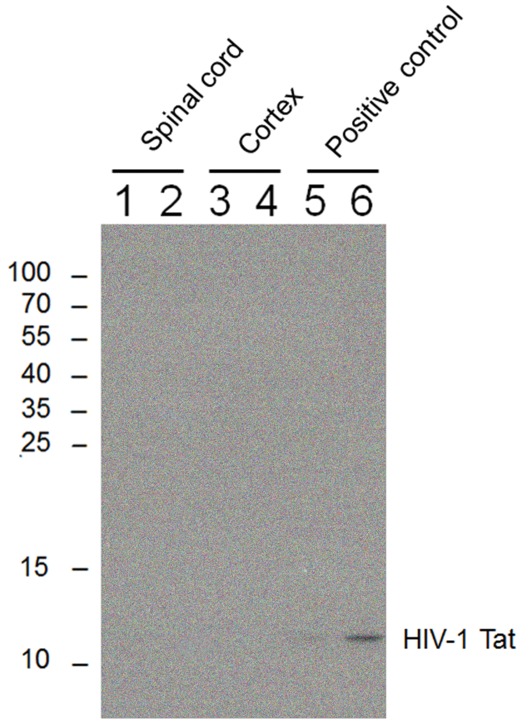

1. Vehicle
2. HIV-1 Tat 100ng/ml
3. Vehicle
4. HIV-1 Tat 100ng/ml
5. HIV-1 Tat 30ng protein
6. HIV-1 Tat 100ng protein

Legends to supplementary figures

Supplementary Figure 1. Intracolonic administration of HIV-1 Tat protein (100 ng/ml) caused a an acute onset diarrhea, with a significant increase of (a) defecation frequency, (b) number of wet spots and (c) fluid accumulation, that were all significantly inhibited by lidocaine (***p<0.001 vs vehicle and lidocaine groups; °°°p<0.001 vs HIV-1 Tat group).

Supplementary Figure 2 To assess recognition memory, object recognition task was performed on different experimental groups at 21 after the administration of HIV-1 Tat. Recognition index (RI) defines the ratio of time spent exploring the novel object over the total time spent exploring both familiar and novel objects. In rats treated with HIV-1 Tat a significant reduction of the RI was observed as compared to vehicle group. Administration of lidocaine restored the novel object RI, while bisacodyl did not significantly affect the novel object RI (*p<0.05 vs vehicle-treated rats; n=8 for each group).

Supplementary Figure 3. Immunoblot analysis showing that following intracolonic administration, HIV-1 Tat protein was virtually absent in the spinal cord (lines 1-2) and the cortex (lines 3-4) homogenates of treated animals; lines 5 and 6, indicate positive controls of HIV-1 Tat protein at 30 and 100 ng, respectively.
